# Association Between Maternal Breastmilk Microbiota Composition and Rotavirus Vaccine Response in African, Asian, and European Infants: A Prospective Cohort Study

**DOI:** 10.1093/infdis/jiad234

**Published:** 2023-06-26

**Authors:** Jonathan Mandolo, Edward P K Parker, Christina Bronowski, Kulandaipalayam Natarajan C Sindhu, Alistair C Darby, Nigel A Cunliffe, Gagandeep Kang, Miren Iturriza-Gómara, Arox W Kamng’ona, Khuzwayo C Jere

**Affiliations:** Virology Research Group, Malawi-Liverpool-Wellcome Trust Clinical Research Programme, Blantyre, Malawi; Department of Clinical Sciences, Liverpool School of Tropical Medicine, Liverpool, United Kingdom; Department of Biomedical Sciences, School of Life Sciences and Allied Health Professions, Kamuzu University of Health Sciences, Blantyre, Malawi; Department of Clinical Research, London School of Hygiene and Tropical Medicine, London, United Kingdom; Department of Clinical Infection, Microbiology and Immunology, Institute of Infection, Veterinary and Ecological Sciences, University of Liverpool, Liverpool, United Kingdom; Wellcome Trust Research Laboratory, Division of Gastrointestinal Sciences, Christian Medical College, Vellore, Tamil Nadu, India; Department of Infection Biology and Microbiomes, Institute of Infection, Veterinary and Ecological Sciences, University of Liverpool, Liverpool, United Kingdom; Department of Clinical Infection, Microbiology and Immunology, Institute of Infection, Veterinary and Ecological Sciences, University of Liverpool, Liverpool, United Kingdom; National Institute for Health and Care Research Health Protection Research Unit in Gastrointestinal Infections, University of Liverpool, Liverpool, United Kingdom; National Institute for Health and Care Research Global Health Research Group on Gastrointestinal Infections, University of Liverpool, Liverpool, United Kingdom; Wellcome Trust Research Laboratory, Division of Gastrointestinal Sciences, Christian Medical College, Vellore, Tamil Nadu, India; Centre for Vaccine Innovation and Access, Program for Appropriate Technology in Health (PATH), Geneva, Switzerland; Virology Research Group, Malawi-Liverpool-Wellcome Trust Clinical Research Programme, Blantyre, Malawi; Department of Biomedical Sciences, School of Life Sciences and Allied Health Professions, Kamuzu University of Health Sciences, Blantyre, Malawi; Virology Research Group, Malawi-Liverpool-Wellcome Trust Clinical Research Programme, Blantyre, Malawi; Department of Clinical Infection, Microbiology and Immunology, Institute of Infection, Veterinary and Ecological Sciences, University of Liverpool, Liverpool, United Kingdom; National Institute for Health and Care Research Health Protection Research Unit in Gastrointestinal Infections, University of Liverpool, Liverpool, United Kingdom; Department of Medical Laboratory Sciences, School of Life Sciences and Allied Health Professions, Kamuzu University of Health Sciences, Blantyre, Malawi

**Keywords:** breastmilk, immunogenicity, microbiota, rotavirus

## Abstract

**Background:**

Maternal breastmilk is a source of pre- and pro-biotics that impact neonatal gut microbiota colonization. Because oral rotavirus vaccines (ORVs) are administered at a time when infants are often breastfed, breastmilk microbiota composition may have a direct or indirect influence on vaccine take and immunogenicity.

**Methods:**

Using standardized methods across sites, we compared breastmilk microbiota composition in relation to geographic location and ORV response in cohorts prospectively followed from birth to 18 weeks of age in India (*n* = 307), Malawi (*n* = 119), and the United Kingdom ([UK] *n* = 60).

**Results:**

Breastmilk microbiota diversity was higher in India and Malawi than the UK across 3 longitudinal samples spanning weeks of life 1 to 13. Dominant taxa such as *Streptococcus* and *Staphylococcus* were consistent across cohorts; however, significant geographic differences were observed in the prevalence and abundance of common and rare genera throughout follow up. No consistent associations were identified between breastmilk microbiota composition and ORV outcomes including seroconversion, vaccine shedding after dose 1, and postvaccination rotavirus-specific immunoglobulin A level.

**Conclusions:**

Our findings suggest that breastmilk microbiota composition may not be a key factor in shaping trends in ORV response within or between countries.

## Teaser

Breastmilk microbiota composition differed significantly between cohorts in India, Malawi, and the UK. No consistent associations were identified between breastmilk microbiota composition and infant oral rotavirus vaccine response.

Breastmilk is a key source of nutrients and contains immunoglobulins (Igs), growth hormones, and oligosaccharides that are critical to infant gut homeostasis and immune development [1]. The *Bifidobacteriaceae*, *Pseudomonadaceae*, *Streptococcaceae*, *Enterococcaceae*, and *Staphylococcaceae* families have consistently been identified as core constituents of the breastmilk microbiota [2–4]. These and other breastmilk taxa may act as a source of commensal bacteria for the developing gut microbiota [5]. Geographic region, delivery mode, and maternal health are among the factors associated with breastmilk microbiota composition [2, 3, 6–9].

More than 100 countries have incorporated oral rotavirus vaccine (ORV) into their national immunization programs [10]. Malawi and the United Kingdom (UK) introduced the live-attenuated G1P[8] Rotarix vaccine into their national immunization programs in 2012 and 2013, respectively [11, 12]. India introduced a live-attenuated G9P[11] vaccine into its immunization program in 2016 [13]. These vaccines have reduced the burden of rotavirus, although in India and Malawi the estimated mortality burden due to rotavirus remained significant as of 2016 (9.2 and 31.2 per 100 000, respectively, compared to 0.1 per 100 000 in England) [14]. The ORV immunogenicity and efficacy is significantly reduced in low- and middle-income countries (LMICs) compared with high-income countries [15]. Given that LMICs account for approximately 95% of rotavirus deaths worldwide [16], the public health burden associated with impaired ORV response is considerable.

Several mechanisms may contribute to the impaired performance of ORV in LMICs. In Malawi and India, we reported infant gut microbiota diversity to be negatively correlated with ORV response [17, 18]. Maternal rotavirus-specific IgG and IgA antibodies in breastmilk and serum were also negatively correlated with ORV response [17, 19], although similar correlations were absent in the UK [17]. Other factors that may impact ORV response include histoblood group antigen status, environmental enteric dysfunction (EED), and prevaccination rotavirus exposure [20].

We hypothesized that breastmilk microbiota composition may be associated with ORV response, either by directly interacting with the vaccine viruses or indirectly via the developing infant gut microbiota. We tested this hypothesis using standardized methods across cohorts in Malawi, India, and the UK [17, 18].

## MATERIALS AND METHODS

### Study Cohort

**Figure 1.**
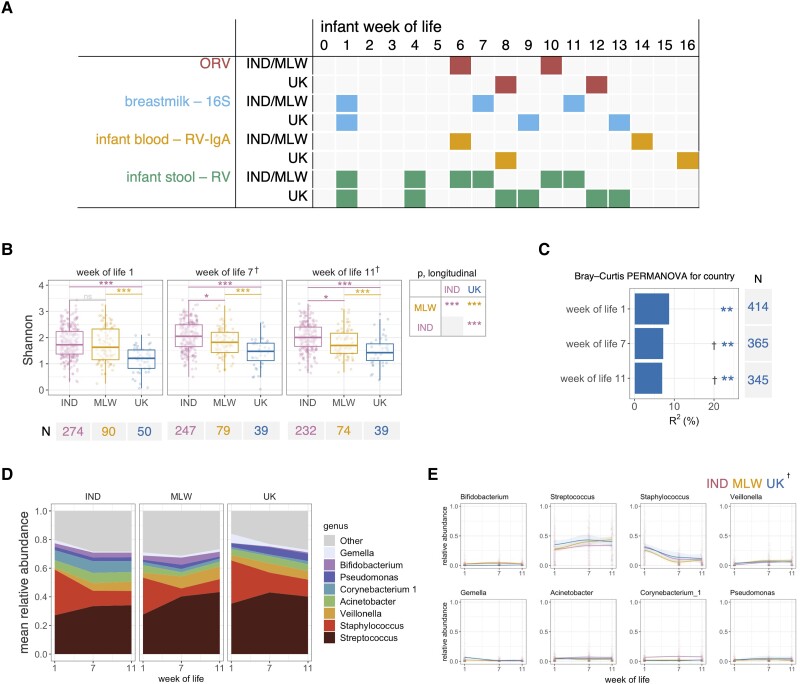
Geographic differences in breastmilk microbiota composition. (*A*) Sample collection strategy. (*B*) Analysis of alpha diversity, based on genus-level Shannon index. Cross-sectional comparisons were performed using analysis of variance (ANOVA) with post hoc Tukey tests. Longitudinal comparisons were performed using mixed-effects regressions with false discovery rate correction of pairwise comparisons. (*C*) Proportion of variation in microbiota composition associated with country, calculated via permutational multivariate ANOVA (PERMANOVA) using genus-level unweighted Bray-Curtis distances. (*D*) Longitudinal plot of mean genus abundances. Genera are included if present with a mean relative abundance of ≥5% in at least 1 country at 1 or more timepoints. (*E*) Longitudinal relative abundance plots for major genera by country. Lines show local weighted regression (loess) fits with 95% confidence intervals. †, +2 weeks for samples collected at weeks of life 7 and 11 in the United Kingdom (UK) due to later vaccination schedule; *P < .05, **P = .001, and ***P < .0005. IND, India; MLW, Malawi; ns, not significant.

This is a follow up to the Rotavirus Vaccine Immunogenicity (RoVI) study—a multisite cohort study exploring the impact of maternal antibodies, microbiota development, and EED on ORV response (CTRI/2015/11/006354). The study design, sample handling, laboratory assays, and primary outcomes of the study have been described previously [17, 18]. Briefly, pregnant women were recruited across sites in Blantyre (Malawi), Vellore (India), and Liverpool (UK). Infants received routine immunizations including 2 doses of Rotarix according to the national immunization schedule at each study site (weeks of life 6 and 10 in India and Malawi; weeks of life 8 and 12 in the UK). Rotavirus-specific IgA (RV-IgA) was measured in infant blood samples collected prevaccination and 4 weeks postvaccination. Rotavirus shedding was measured in 6 longitudinal stool samples per infant, including 1 week after each ORV dose. Breastmilk samples were collected in week of life 1 and 1 week after each ORV dose (Figure 1*A*).

### Sample Processing

Breastmilk samples were collected in sterile sample pots by participants and transferred to the site-specific laboratory by courier within 24 hours (refrigerated throughout) in the UK or within 4 hours in India and Malawi. Upon receipt, samples were kept at 4°C for a maximum of 8 hours until processing and subsequently stored in 2-mL aliquots in SuperLock tubes (Starlab) at −70°C for a maximum of 2 weeks before deoxyribonucleic acid (DNA) extraction. The DNA was extracted from 1 mL of breastmilk but otherwise followed the stool-specific protocol previously described [17]. A negative extraction control was included in each extraction batch. The DNA extracts from Malawi and India were shipped on dry ice to the University of Liverpool for library preparation and sequencing.

### Microbiota Sequencing

Breastmilk microbiota composition was determined by sequencing the 16S rRNA gene V3–V4 region. Amplicon generation, library preparation, and sequencing were performed as previously described for stool [17], but with 15 cycles (vs 10) for the initial amplicon polymerase chain reaction (PCR) and 20 cycles (vs 15) for subsequent indexing PCR to ensure robust amplification from the low-biomass samples. We sequenced amplicons for 1301 separate breastmilk samples (894 from India, 275 from Malawi, and 132 from the UK) across 6 Illumina HiSeq2500 lanes (v2 chemistry with 600 cycles in rapid run mode). Samples from each participant were processed on the same plate. Sequencing was batched by geographic location according to sample availability. Each PCR plate included the following: a no-template PCR control; a breastmilk control sample provided by a mother in the UK who was not enrolled in the study; DNA from a mock community (Zymo Research D6306); and a pool of extraction controls corresponding to the samples contained on each plate. Due to shipment challenges, extraction controls corresponding to 141 of 243 (58%) of the samples from Malawi were included in the extraction pools. To better define the amplicon profile of extraction controls, we sequenced an additional 49 pools containing 1–5 controls from extraction batches performed in India or the UK. Final libraries contained up to four 96-well PCR plates (384 amplicons). Breastmilk DNA samples were amplified on separate plates to stool samples, although we allowed mixing of stool and breastmilk PCR plates in a given library. To validate the robustness of the sequencing protocol, 90 breastmilk DNA samples (30 per cohort, all collected in week of life 1) were transferred to Imperial College London and sequenced according to the methods above with minor modifications, as previously described [17].

### Bioinformatic Processing

Adapters were trimmed from raw sequences using cutadapt version 1.18 [21]. We merged, filtered, and denoised the amplicon sequences using the DADA2 pipeline in QIIME2 (version 2018.11) [22]. Forward and reverse reads were truncated to 270 and 200 base pairs (bp), respectively. Taxonomic assignment was performed via the *dada2* package (version 1.14.1) using the Ribosomal Database Project (RDP)-naive Bayesian classifier trained on the Silva rRNA database (version 132). Additional data management and filtering steps were performed using the *phyloseq* package in R (version 1.38.0). Ribosomal sequence variants (RSVs) were retained if they were 390–440 bp in length (given an amplicon length distribution after primer trimming with peaks at 400–410 bp and 420–430 bp), assigned as bacterial, detectable at ≥0.1% abundance in at least 1 sample, and passed frequency-based contamination filtering using the *decontam* package in R (version 3.6.1) [23]. Nanodrop readings (nanogram/microliter) were used to define concentration of the input template.

Given the additional amplification involved in library preparation for breastmilk samples, reads were frequently detected in extraction controls (*n* = 56 individual or pooled controls with >10 000 reads after the filtering steps above). Several additional filtering steps were therefore included. First, we retained RSVs if they were detectable at ≥0.1% abundance in ≥1% of breastmilk samples from at least 1 country. Second, we applied prevalence-based filtering using the *decontam* package with a *P* value threshold of .05 to exclude RSVs that were more common in extraction controls. Finally, we removed samples if their mean Bray-Curtis distance (based on either weighted or unweighted metrics) from breastmilk extraction controls was smaller than their mean distance from other breastmilk samples collected from the same country (Supplementary Figure 1).

### Outcomes

We compared breastmilk microbiota composition by country and ORV response. Our primary indicator of ORV response was seroconversion status—defined as a 4-fold increase in RV-IgA concentration or detection of antibodies at ≥20 IU/mL in previously seronegative infants. Secondary outcomes included postvaccination RV-IgA concentration and rotavirus shedding 1 week after the first dose of ORV (as an indicator of vaccine virus take). Shedding was detected via real-time PCR targeting the Rotarix *NSP2* gene [24]. We also performed an exploratory analysis of alpha and beta diversity to identify demographic and clinical factors associated with breastmilk composition.

### Statistical Analysis

Analyses were performed in the programming language R using the statistical pipeline previously described for stool samples with minor modifications [17]. Alpha and beta diversity were calculated at a rarefaction depth of 15 000 sequences per sample. We performed cross-sectional analyses of alpha diversity via analysis of variance (ANOVA), logistic regression (binary ORV outcomes), Pearson's r with 2-sided hypothesis testing (log-transformed RV-IgA), and linear regression (exploratory covariates). Pearson's r was also used to compare alpha diversity in paired breastmilk and infant stool samples, with data for the latter obtained as previously described [17]. We assessed beta diversity using permutational multivariate ANOVA (PERMANOVA) with 999 permutations based on genus-level unweighted Bray­-Curtis distances. For binary outcomes, discriminant genera and RSVs were identified via 2-sided Fisher's exact test (differences in prevalence) and Aldex2 (2-sided Wilcoxon rank-sum test of centred log-ratio transformed sequence counts), with taxa classified as discriminant if they had a *P* < .05 based on either method after Benjamini-Hochberg false discovery rate (FDR) adjustment. Aldex2 was used to identify taxa correlated with log-transformed RV-IgA (FDR-adjusted *P* < .05 based on 2-sided Spearman's rank test). Taxa were included in the analysis if they were detected with a prevalence of >5% in at least one of the groups being compared. We supplemented cross-sectional analyses with longitudinal mixed-effects models of Shannon index and taxon abundances (zero-inflated negative binomial models of genus-level sequence counts), including week of life as a covariate and study identification (ID) as a random effect. Genera were included in longitudinal models if they were present in 20% of samples in a given country.

We applied Random Forests to predict country and ORV outcome based on genus or RSV relative abundances. For each analysis, we performed 20 iterations of 5-fold cross-validation. For binary outcomes, we standardized the baseline accuracy at 50% by fitting each iteration on a random subset of 50 samples per group (or the number of samples in the minority group if this was <50). Models were excluded if there were <10 samples in the minority group. For regression models, accuracy was quantified using linear regression to determine the out-of-bag R^2^ values for predicted versus observed RV-IgA values. For positive controls and technical replicates, we used linear regression (alpha diversity and common genera abundances) and PERMANOVA (beta diversity) to quantify the proportion of variance explained by sample ID.

Raw sequence data for this study have been deposited in the European Nucleotide Archive (accession code PRJEB38948). Processed data and analysis code are available on Github (https://github.com/eparker12/RoVI).

### Ethics Approval

The study was approved by the Institutional Review Board (IRB) at the Christian Medical College in Vellore (IRB No. 9472/24.06.2015), the College of Medicine Research and Ethics Committee in Blantyre (P.01/16/1853), and the North West - Liverpool Central Research Ethics Committee in Liverpool (15/NW/0924).

## RESULTS

### Study Cohort

Overall, 664 mother-infant pairs (395 in India, 187 in Malawi, and 82 in the UK) were enrolled in the study, and the primary endpoint (measurement of seroconversion or dose 1 shedding) was reached for 484 (307 in India, 119 in Malawi, and 60 in the UK). Baseline characteristics, wild-type rotavirus infection status, EED biomarker levels, and infant stool microbiota composition have previously been compared by country and ORV outcome [17]. Exclusive breastfeeding was reported by 265 of 307 (86%) mothers in India, 108 of 119 (91%) in Malawi, and 26 of 60 (43%) in the UK, with partial breastfeeding reported by a further 32 of 307 (10%) in India, 11 of 119 (9%) in Malawi, and 20 of 60 (33%) in the UK. Exclusive breastfeeding was positively correlated with ORV seroconversion and postvaccination infant RV-IgA levels in India but not in other cohorts. Breastmilk RV-IgA levels were negatively correlated with infant RV-IgA levels in India and Malawi [17].

### Oral Rotavirus Vaccine Shedding and Immunogenicity

As previously reported [17], seroconversion was observed in 27 of 51 (53%) infants in the UK, 85 of 305 (28%) in India, and 24 of 103 (23%) in Malawi. Rotavirus shedding 1 week after the first dose of ORV was detected in 55 of 60 (92%) infants in the UK, 82 of 305 (27%) in India, and 56 of 101 (55%) in Malawi. Geometric mean concentrations of RV-IgA (IU/mL) after vaccination were 27 (17–45) in the UK, 20 (95% CI 16–25) in India, and 9 (6–12) in Malawi.

Indian infants were characterized by high rates of neonatal rotavirus infection, defined as detection of wild-type rotavirus shedding in week 1 of life or baseline seropositivity (prevaccination RV-IgA ≥20 IU/mL). This was observed in 166 of 304 (55%) infants in India, 10 of 90 (11%) in Malawi, and 2 of 54 (4%) in the UK. Given the potential impact of neonatal infection on ORV response [17], we report results for the Indian cohort overall and stratified by neonatal infection status below.

### Geographic Differences in Breastmilk Microbiota Composition

Of 1301 breastmilk samples sequenced, 1124 yielded high-quality microbiota profiles (≥15 000 sequences after quality filtering; 95 075 ± 113 894 [mean ± standard deviation] sequences per sample). Microbiota profiles of positive controls and technical replicates were consistent across sequencing runs and facilities (Supplementary Figure 2).

There were marked differences in breastmilk microbiota composition between cohorts. Microbiota diversity was significantly lower in UK than both other cohorts at all timepoints. Diversity was similar in India and Malawi at week of life 1, but it was higher in India than Malawi at weeks of life 7 and 11 (Figure 1*B*). Samples were clustered by individual (PERMANOVA R^2^ = 49%, *P* = .001), with country accounting for 7%–9% of variation depending on age (Figure 1*C*). Although 350 genera were detected overall, a small proportion were dominant in each cohort (Figure 1*C* and Supplementary Figure 3). Among dominant genera, *Streptococcus* was depleted in India compared with both other cohorts, whereas *Acinetobacter* and *Corynebacterium* were enriched (FDR *P* < .05 based on longitudinal models) (Supplementary Figure 4). *Staphylococcus* followed a parallel trajectory in each cohort, peaking in week of life 1, and it was less abundant in Malawi than both of the other cohorts. *Bifidobacterium* was less abundant in the UK, reflecting the pattern previously reported for stool samples [17], whereas *Gemella* was enriched in this cohort (Figure 1*D*). Several of these discrepancies in dominant genera were also evident in cross-sectional analyses of prevalence and/or abundance (Supplementary Table 1).

Additional discriminant taxa detected by longitudinal and cross-sectional models are reported in Supplementary Figure 4 and Supplementary Table 1. Based on longitudinal models, 17 genera were enriched in India compared with both other cohorts, including 9 Proteobacteria (eg, *Aeromonas* and *Alishewenalla*), 3 Firmicutes (eg, *Enterococcus* and *Aerococcus*), and 5 Actinobacteria (eg, *Dermacoccus*). Nine genera were enriched in Malawi compared with both other cohorts, including the Bacteroidetes genus *Prevotalla* 9 alongside 8 Firmicutes (eg, *Faecalibacterium* and *Lachnospiraceae*). Three genera—*Gemella*, *Haemophilus*, and *Enterobacter*—were enriched in the UK compared with the other cohorts.

Random Forests discriminated samples by country with high accuracy (median cross-validation accuracies of 85%–95%; baseline accuracy 50%) (Supplementary Figure 5). Genera underlying model accuracy (based on mean importance scores) were consistent with the discriminant taxa described above (Supplementary Table 1).

**Figure 2.**
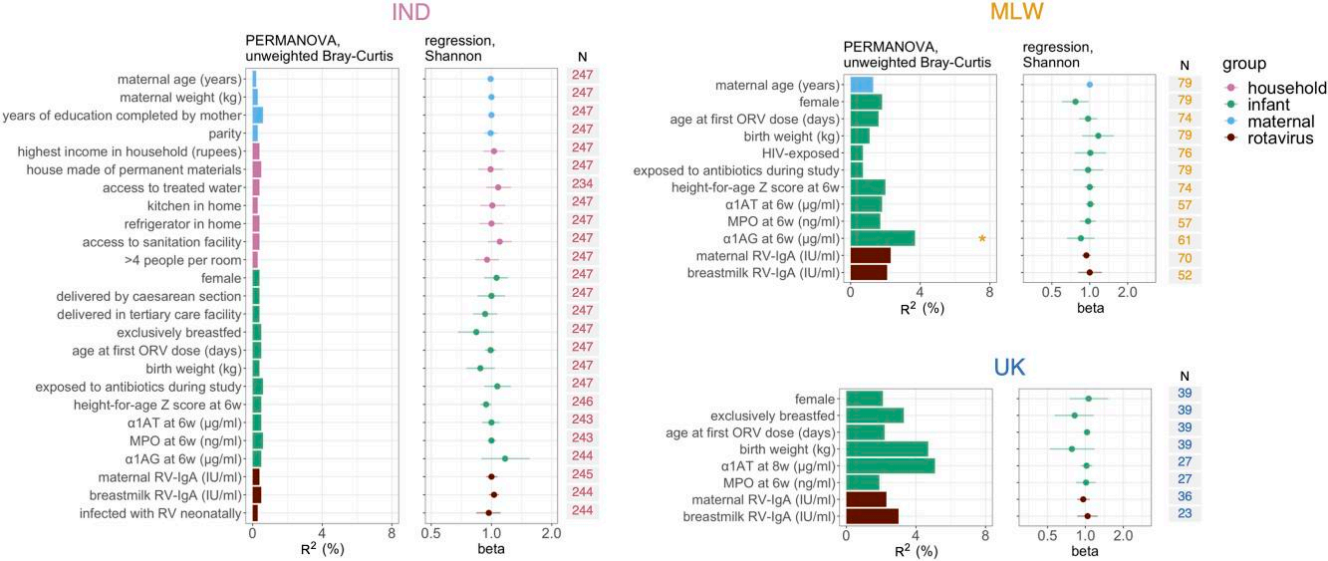
Cofactors associated with breastmilk microbiota composition. Samples collected 1 week after the first dose of oral rotavirus vaccine were included (week of life 7 in India and Malawi; week of life 9 in the United Kingdom [UK]). The left panel, presenting data for Indian samples (n = 247), contains the full list of exploratory variables (with the exception of human immunodeficiency virus exposure status, which was also assessed for Malawi). For analyses of samples from Malawi and the UK (right panels; n = 79 and 39, respectively), variables were excluded if they were not measured or exhibited limited variability (n < 10 in either comparison group). Permutational multivariate analysis of variance (PERMANOVA) was performed using genus-level unweighted Bray-Curtis distances. Shannon index was calculated at genus level and assessed as an outcome variable via linear regression. *False discovery rate (FDR) *P* < .05. Α1AT, α1-antitrypsin; α1AG, α1 acid glycoprotein; IND, India; MLW, Malawi; MPO, myeloperoxidase; ORV, oral rotavirus vaccine; RV, rotavirus.

We also assessed alpha and beta diversity of breastmilk samples in relation to individual-level variables measured in each cohort (Figure 2). With the exception of infant serum α1 acid glycoprotein level (a marker of systemic inflammation), which was modestly associated with beta diversity in Malawian samples (R^2^ = 3.7%), no covariates were significantly associated with breastmilk microbiota composition. Alpha diversity in paired breastmilk and infant stool samples was not significantly correlated at week of life 1 or at the time of either ORV dose in any cohort (Supplementary Figure 6).

### Breastmilk Microbiota Composition Versus Oral Rotavirus Vaccine Response

**Figure 3.**
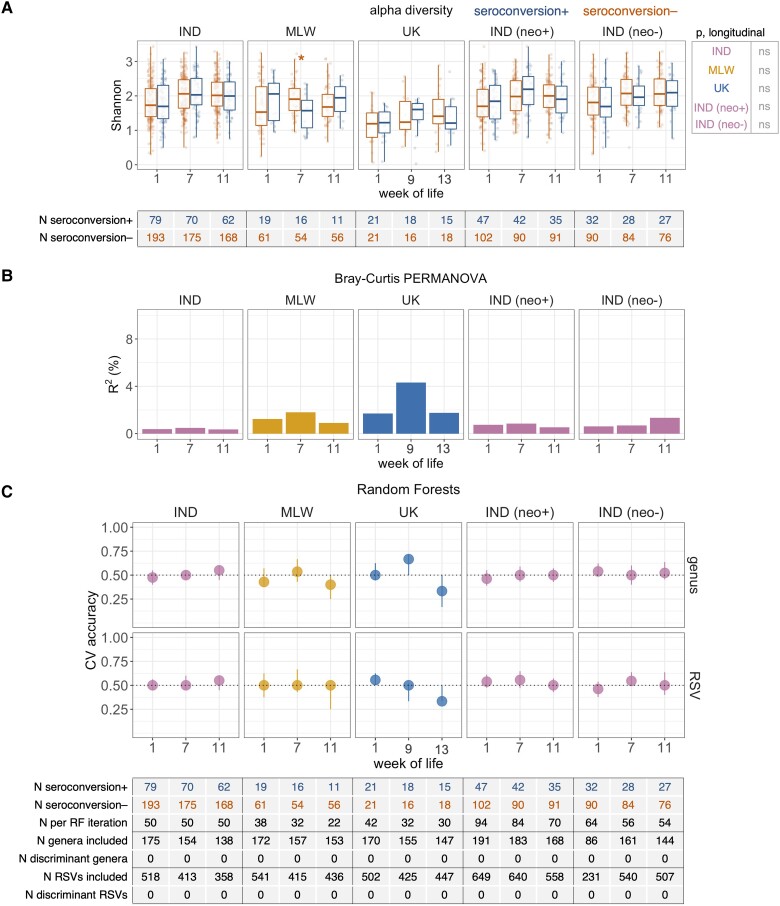
Association between breastmilk microbiota composition and oral rotavirus vaccine seroconversion. (*A*) Analysis of alpha diversity, based on genus-level Shannon index. Cross-sectional comparisons were performed using logistic regression. Longitudinal comparisons were performed using mixed-effects models. (*B*) Proportion of variation in microbiota composition associated with seroconversion, calculated via permutational multivariate analysis of variance (PERMANOVA) using genus-level unweighted Bray-Curtis distances. (*C*) Cross-validation (CV) accuracy of Random Forests for prediction of seroconversion. Median out-of-bag accuracy (proportion correctly assigned) and interquartile range across 20 iterations of 5-fold CV are displayed. Each iteration included an equal number of responders and nonresponders (50 per group where possible, or else the number in the minority group if this was <50). Taxa were classified as discriminant if they had a false discovery rate (FDR)-adjusted *P* < .05 based on either 2-sided Fisher's exact test (differences in prevalence) or Aldex2 with 2-sided Wilcoxon rank-sum test (differences in abundance). *P < .05. IND, India; MLW, Malawi; neo+, infected with rotavirus neonatally (defined by detection of rotavirus shedding in week of life 1 or baseline seropositivity); neo−, uninfected with rotavirus neonatally; ns, not significant; RF, Random Forests; RSV, ribosomal sequence variant.

Based on longitudinal models of Shannon index, we observed no significant differences in microbiota diversity according to seroconversion status in any cohort (Figure 3*A*). This was also the case for cross-sectional analyses, with the exception of comparisons in Malawi at week 7 of life (the week after the first dose of ORV), wherein Shannon index was negatively correlated with seroconversion. Beta diversity analyses based on genus-level unweighted Bray-Curtis distances did not reveal any significant association between breastmilk microbiota composition and seroconversion status (Figure 3*B*). Likewise, Random Forest models based on genus or

RSV abundances failed to accurately predict seroconversion (Figure 3*C*), and no discriminant taxa were identified based on cross-sectional analyses of prevalence or abundance after FDR correction. Longitudinal models of common genera (≥20%) revealed frequent age-associated changes in taxon abundance but only 1 significant association with seroconversion (a negative correlation between *Alloprevotella* abundance and seroconversion in Malawi) (Supplementary Table 2).

Cross-sectional analyses of secondary ORV endpoints, including postvaccination RV-IgA concentration (Supplementary Figure 7) and dose 1 ORV shedding (Supplementary Figure 8), mirrored those for seroconversion, revealing no consistent associations. In the UK, beta diversity at week of life 9 was modestly associated with postvaccination RV-IgA (R^2^ = 6%, *P* = .005), but this was not the case for any other timepoint or in other cohorts. Very few discriminant genera were identified with respect to these secondary outcomes based on longitudinal models of genus abundance (Supplementary Table 2).

## DISCUSSION

Breastmilk is a key source of pre- and pro-biotics that shape infant gut microbiota configuration and immune development. We documented significant differences in breastmilk microbiota composition between Malawi, India, and the UK. However, no consistent differences in breastmilk microbiota composition were observed with respect to ORV response.

Despite the geographic differences in microbiota composition, several genera were dominant across the 3 cohorts. Together, *Streptococcus*, *Staphylococcus*, *Acinetobacter*, *Bifidobacterium*, *Veillonella*, *Gemella*, *Corynebacterium*, and *Pseudomonas* formed approximately 75% of the breastmilk microbiota—consistent with the dominant taxa reported elsewhere [2–4, 25]. The relative abundances of these dominant genera changed over time, with *Staphylococcus* declining in abundance while *Streptococcus* and *Veillonella* increased. This is similar to the trajectory in breastmilk microbiota composition reported in Kenya [26]. The infant salivary microbiota is known to be colonized by *Streptococcus* [27, 28], such that the continued dominance of *Streptococcus* in maternal breastmilk may partly reflect breastmilk-saliva interplay. Skin-associated genera including *Staphylococcus* and *Corynebacterium* were also among the dominant genera in maternal breastmilk, consistent with previous findings [25].

Breastmilk microbiota diversity was higher in Malawi and India than the UK. This contrasts with discrepancies we reported in stool microbiota diversity, which was higher in Malawi than both India and the UK at week 1 of life but converged over the ensuing 6–8 weeks [17]. In a previous cross-sectional study spanning 11 sites, breastmilk microbiota diversity was highest in rural Ethiopia and lowest in Ghana, with intermediate levels across other sites in Africa, Europe, North America, and South America [8]. We observed *Streptococcus* to be more abundant in Malawi and the UK than in India, whereas *Bifidobacterium* was depleted in the UK compared with both other cohorts. Prior studies have also highlighted geographically distinct abundance profiles including depletion of *Bifidobacterium* in European compared with African samples [8]. Together, these studies highlight the significant regional variation that occurs in breastmilk microbiota diversity and composition. To delineate overarching global trends (eg, urban vs rural, high-income vs LMIC), future studies integrating representative data from multiple countries, such as the present, are warranted.

We did not observe consistent associations between breastmilk microbiota composition and ORV response. At the time of the first ORV dose, breastmilk microbiota diversity in Malawi was negatively correlated with ORV seroconversion—a correlation that was also apparent among infant stool samples in this cohort [17]. Whereas we reported consistent correlations between diversity and seroconversion among Indian and Malawian infants’ stool samples, there was no consistent discrepancy across cohorts in breastmilk. Moreover, we did not observe a significant correlation between the microbiota diversity of paired breastmilk and infant stool samples, suggesting that the negative correlations between microbiota diversity and ORV seroconversion in Malawi are not causally related. A previous study in India documented higher *Enterobacter/Klebsiella* abundance in breastmilk and infant stool samples of infants with symptomatic rotavirus disease compared to those with asymptomatic or no infection [29]. However, no significant discrepancies were observed between neonates with asymptomatic infection and those lacking infection, which is consistent with the lack of association reported here in relation to attenuated viral exposure via ORV.

To our knowledge, this is the first study to explore the link between breastmilk microbiota composition and ORV response. Our study is strengthened by the use of standardized methods across cohorts, including multiple indicators of ORV response. Nonetheless, several limitations of the present study should be considered. Breastmilk samples were collected 1 week after each ORV dose, so they may offer an imperfect proxy for microbiota composition at the time of vaccination. Owing to recruitment challenges in Malawi [17], we fell short of the target sample size in this cohort (*n* = 119 rather than 150), potentially undermining our ability to detect relevant associations in this cohort. Because of their low biomass, breastmilk samples were subjected to extra rounds of amplification to attain adequate material for sequencing, leading to amplification from extraction controls. We accounted for this via stringent filtering of contaminants. Nonetheless, the potential contribution of contamination and site-specific batch effects to the observed trends cannot be discounted.

## CONCLUSIONS

Our findings suggest that breastmilk microbiota composition may not be a key factor shaping ORV response within or between countries. Other components of human milk would be a valuable focus of future investigation. Human milk oligosaccharides such as lacto-*N*-tetraose have previously been linked with symptomatic rotavirus infection in Indian neonates, possibly via an effect on neonatal G10P [11] rotavirus infectivity [29]. Future studies of the breastmilk metabolome may help discern whether similar factors influence the immunogenicity and efficacy of ORV.

## Supplementary Data

Supplementary materials are available at *The Journal of Infectious Diseases* online. Consisting of data provided by the authors to benefit the reader, the posted materials are not copyedited and are the sole responsibility of the authors, so questions or comments should be addressed to the corresponding author.

## Supplementary Material

jiad234_Supplementary_Data

## Notes

***Acknowledgments.*** We thank the following: all members of the clinical study teams in Vellore, Blantyre, and Liverpool, including Falak Diab, Siobhan Holt, and the research midwives at the Liverpool Women's Hospital; Dawn Redman and the team of research nurses at Alder Hey Children's Hospital; Uma Raman, Charlet, Margaret, Jacklin, and the field research assistants at Christian Medical College, Vellore; and James Tamani, Anna Ainani, Amisa Chisale, Bertha Masamba, Carlo Gondwe, and Evelyn Gondwe in Blantyre, Malawi. Richard Eccles, Anita Lucaci, Richard Gregory, John Kenny, and other staff at the Centre for Genomic Research (University of Liverpool) provided valuable support for the 16S microbiota sequencing work. We are grateful to all of the families involved in the study.

***Author contributions.*** MI-G, ACD, EPKP, KCJ, AWK, and GK contributed to conceptualization; JM, CB, EPKP, ACD, MI-G, and GK contributed to methodology; EPKP contributed to software and validation; JM and EPKP contributed to formal analysis; JM, CB, EPKP, AWK, and KCJ contributed to investigation; CB, EPKP, and JM contributed to data curation; JM and EPKP contributed to writing the original draft; ACD, NAC, MI-G, AWK, and KCJ contributed to review and editing; EPKP and JM contributed to visualization; NAK and KCJ contributed to supervision; CB, MI-G, KNS, KCJ, and GK contributed to project administration; MI-G and GK contributed to funding acquisition. All authors read and approved the manuscript.

***Disclaimer.*** The funders had no role in the study design, data collection and interpretation, or the decision to submit the work for publication. The authors received no financial support or other form of compensation related to the development of the manuscript. The views expressed are those of the author(s) and not necessarily those of the National Institute for Health and Care Research (NIHR), the Department of Health and Social Care, or the UK Health Security Agency (UKHSA).

***Financial support*.** This work was supported by different funders per site. The UK and Malawi sites were funded by the UK Medical Research Council and the UK Department for International Development (Newton Fund MR/N006259/1). KCJ is funded by a Wellcome Trust International Training Fellowship (number 201945/Z/16/Z). The site in India was funded by the Department of Biotechnology, Ministry of Science and Technology, India.
