# Supplementary material for: Association Between Maternal Breastmilk Microbiota Composition and Rotavirus Vaccine Response in African, Asian, and European Infants: A Prospective Cohort Study
Source: J Infect Dis. 2023 Jun 26;228(5):637–45. doi: 10.1093/infdis/jiad234 (PMC10469347; doi:10.1093/infdis/jiad234)
Supplement: jiad234_Supplementary_Data [file jiad234_supplementary_data.zip › RoVI BM supplementary figures, 13-03-2023.pdf]

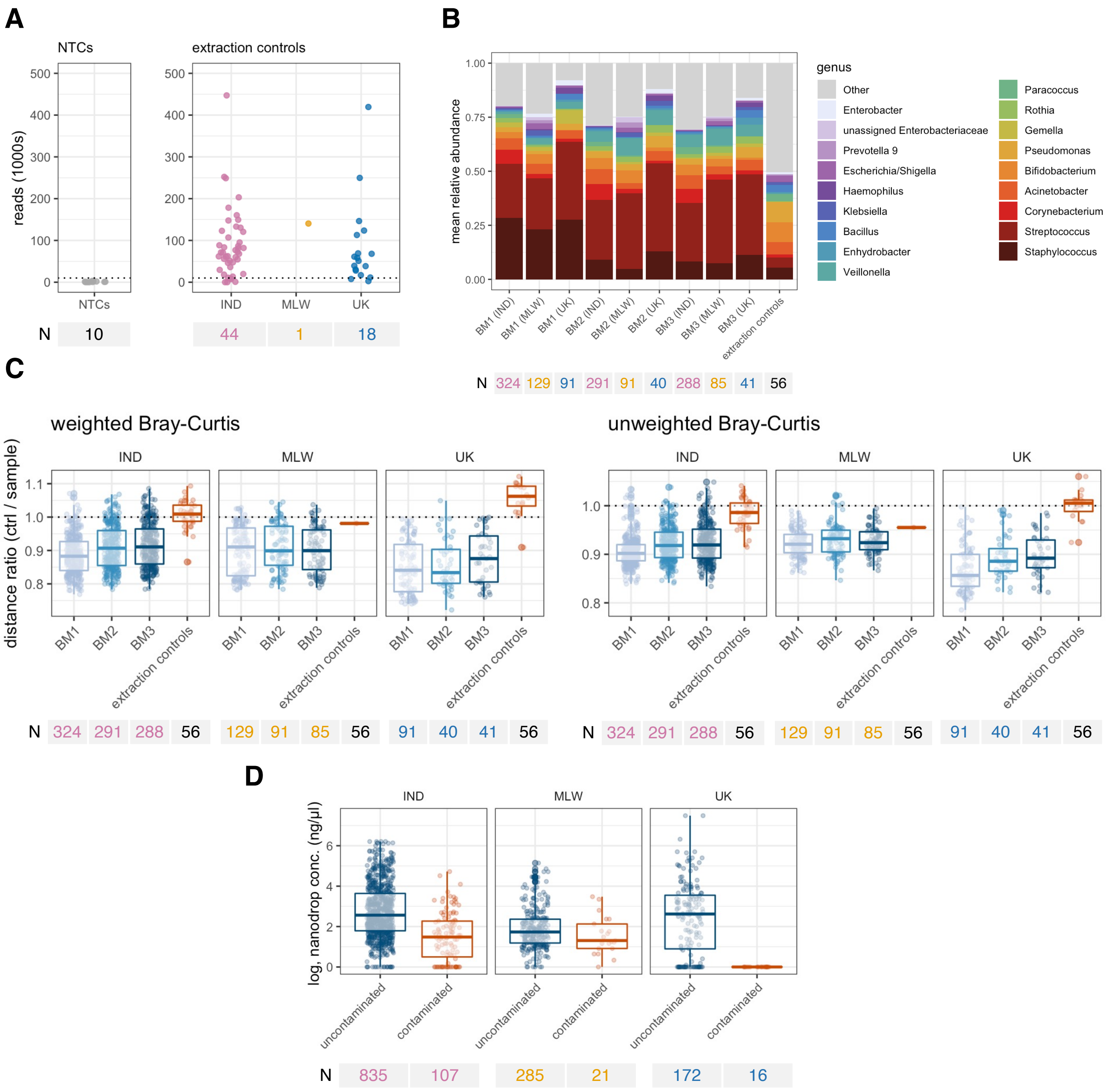

Supplementary Figure 1. Contaminant filtering steps for breastmilk samples. (A) Read counts for negative controls. Counts from were consistently above 10,000 (dotted line) for pooled or individual extraction controls but not NTCs. **(B)** Mean genus abundance profile by sample type and country. Extraction controls displayed a distinct genus abundance profile with notable enrichment of rare taxa (labelled 'other'). Samples or controls with at least 10,000 sequences were included. **(C)** Identification of samples with a contaminant profile. For each sample, the mean distance was calculated from other breastmilk samples from the same country and all breastmilk extraction controls. If the sample clusters more closely with other samples on average, the ratio of these distances will be <1. **(D)** Nanodrop concentrations of samples identified as potentially contaminated based on either weighted or unweighted Bray-Curtis (ratio >1 in panel [**C**]). A pseudocount of 1 was added before log transformation.

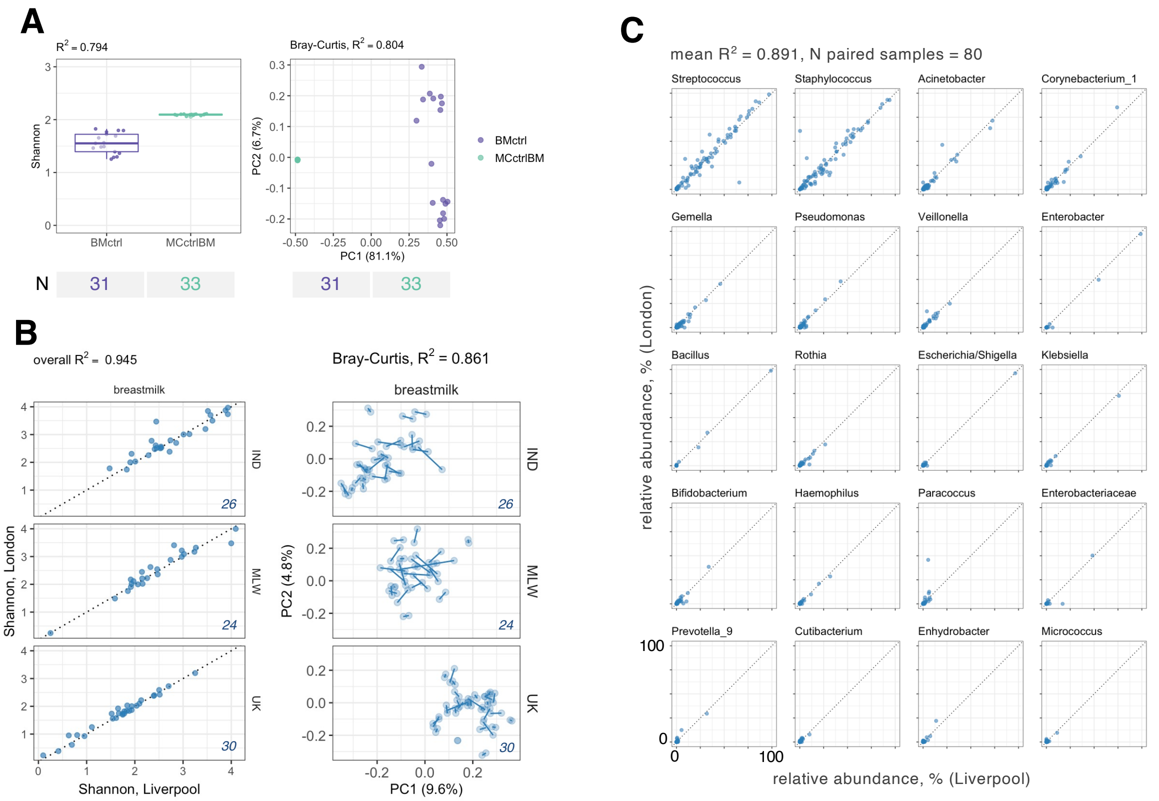

Supplementary Figure 2. Technical replicate profile. Alpha diversity and beta diversity for **(A)** positive controls and **(B)** validation replicates at ribosomal sequence variant level. Positive controls were included on each PCR plate and included a breastmilk sample (BMctrl) and a mock bacterial community (MCctrl). Validation samples were processed at a separate sequencing facility. These were evenly distributed across the study sites (30 per site per sample group) and randomised across a single sequencing plate. Sample pairs were retained in the analysis of both technical replicates had ≥15,000 sequences after quality filtering (80/90 [89%]). The proportion of variation attributable to sample type was determined by linear regression (for Shannon index) and PERMANOVA of unweighted Bray–Curtis distances (for beta diversity). In **(B),** technical replicates are linked by a line in the right-hand panel. Sample counts are indicated in italics. **(C)** Relative abundances of major genera in validation samples. R^2 was determined based on linear regression.

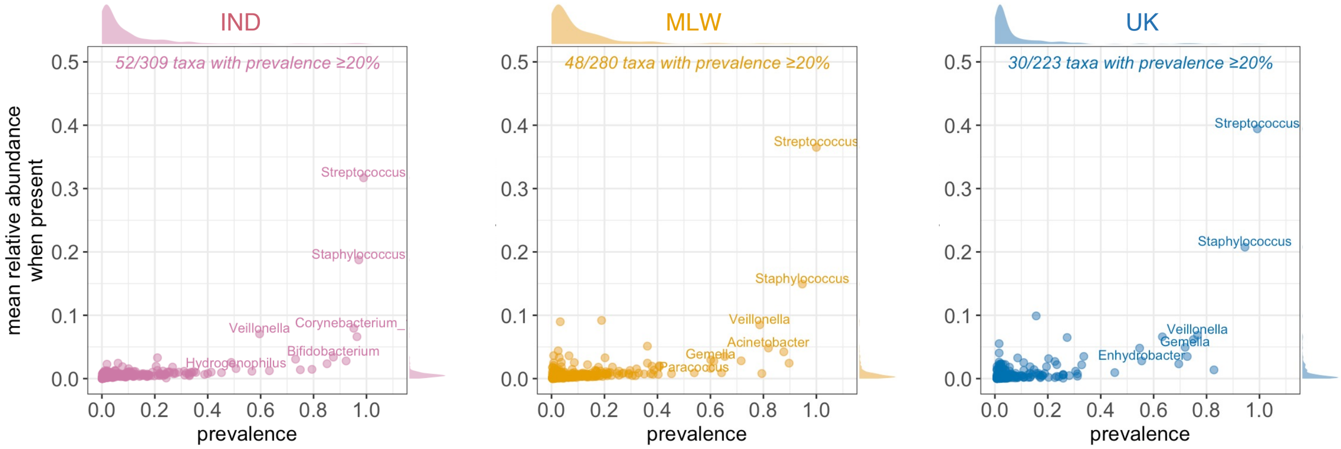

Supplementary Figure 3. Genus abundance and prevalence profile of breastmilk samples. All breastmilk samples from each cohort were included in the prevalence and abundance calculations (n = 753, 243, and 128 for India, Malawi, and the UK, respectively). Margins display density plots. IND, India; MLW, Malawi.

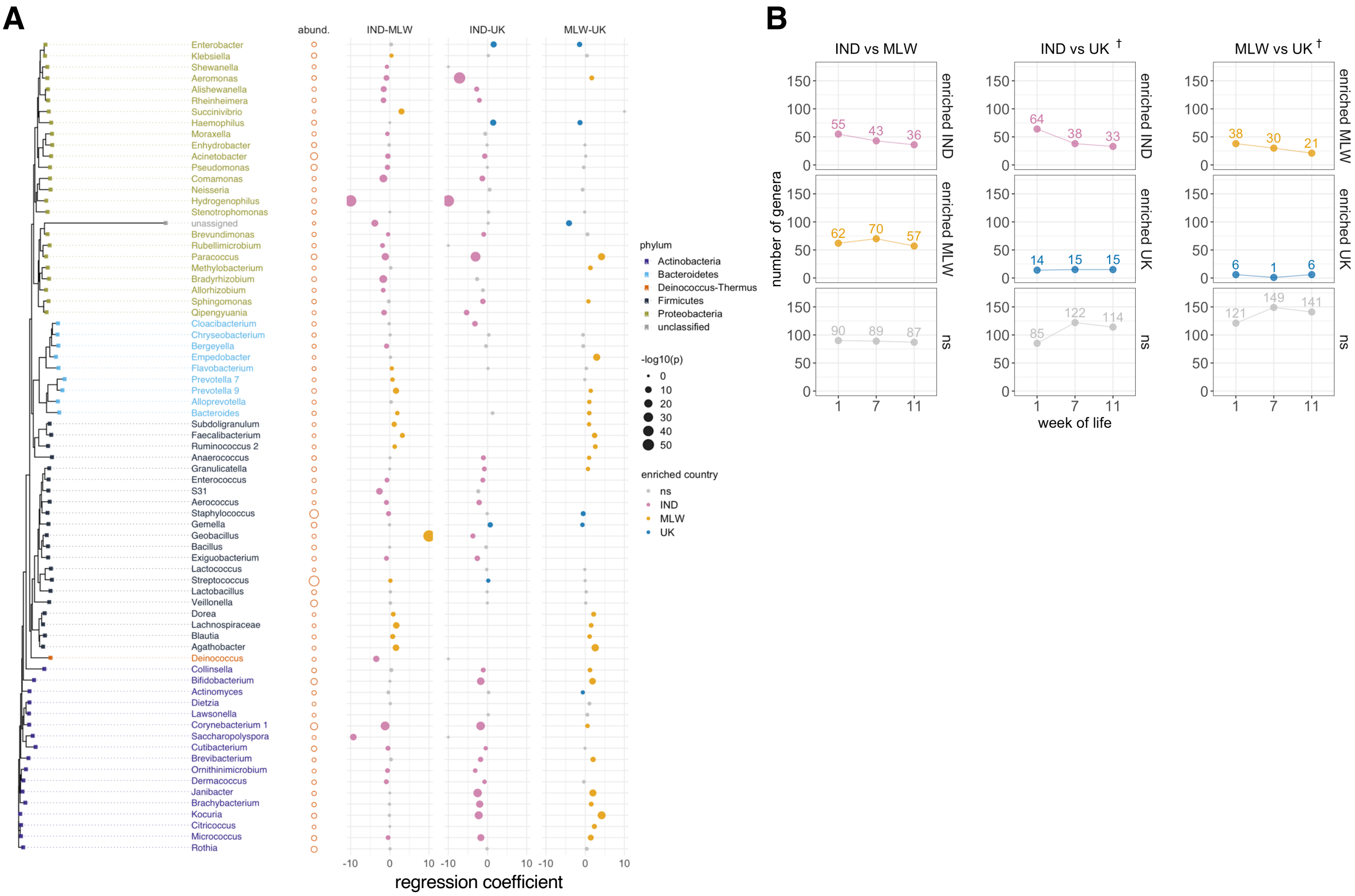

C^1	IND	IND	MLW
N^1 samples (N^1 infants)	753 (302)	753 (302)	243 (108)
C^2	MLW	UK	UK
N^2 samples (N^2 infants)	243 (108)	128 (51)	128 (51)
N taxa tested	70	58	54
enriched IND (FDR p<0.05)	29	28	–
enriched MLW (FDR p<0.05)	15	–	26
enriched UK (FDR p<0.05)	–	4	6

Supplementary Figure 4. (A) Longitudinal models of genus abundance by country. Mixed-effects zero-inflated negative binomial models regressions were used to identify discriminant genera. Genera were included if present in at least 20% of samples from at least one country being compared. Regression coefficients are displayed with point size scaled by p value. Genus order is based on a neighbour-joining tree derived from JC69 distances, with the most abundant ribosomal sequence variant serving as the reference sequence for each genus. Circles to the right of the tree are scaled by mean relative abundance across infant samples (following arcsine square root transformation). **(B)** Cross-sectional comparisons of genus abundance by country. Discriminant genera were identified based on two-sided Fisher's exact test (differences in prevalence) and Aldex2 with two-sided Wilcoxon rank sum test (differences in abundance). The number of genera with an FDR-adjusted p value of <0.05 based on either method is highlighted for each pairwise cross-sectional comparison. C, country; FDR, false discovery rate; IND, India; MLW, Malawi; ns, not significant; †, +2 weeks samples collected at weeks of life 7 and 11 in the UK due to later vaccination schedule. See **Supplementary Table 1** for full details of discriminant taxa.

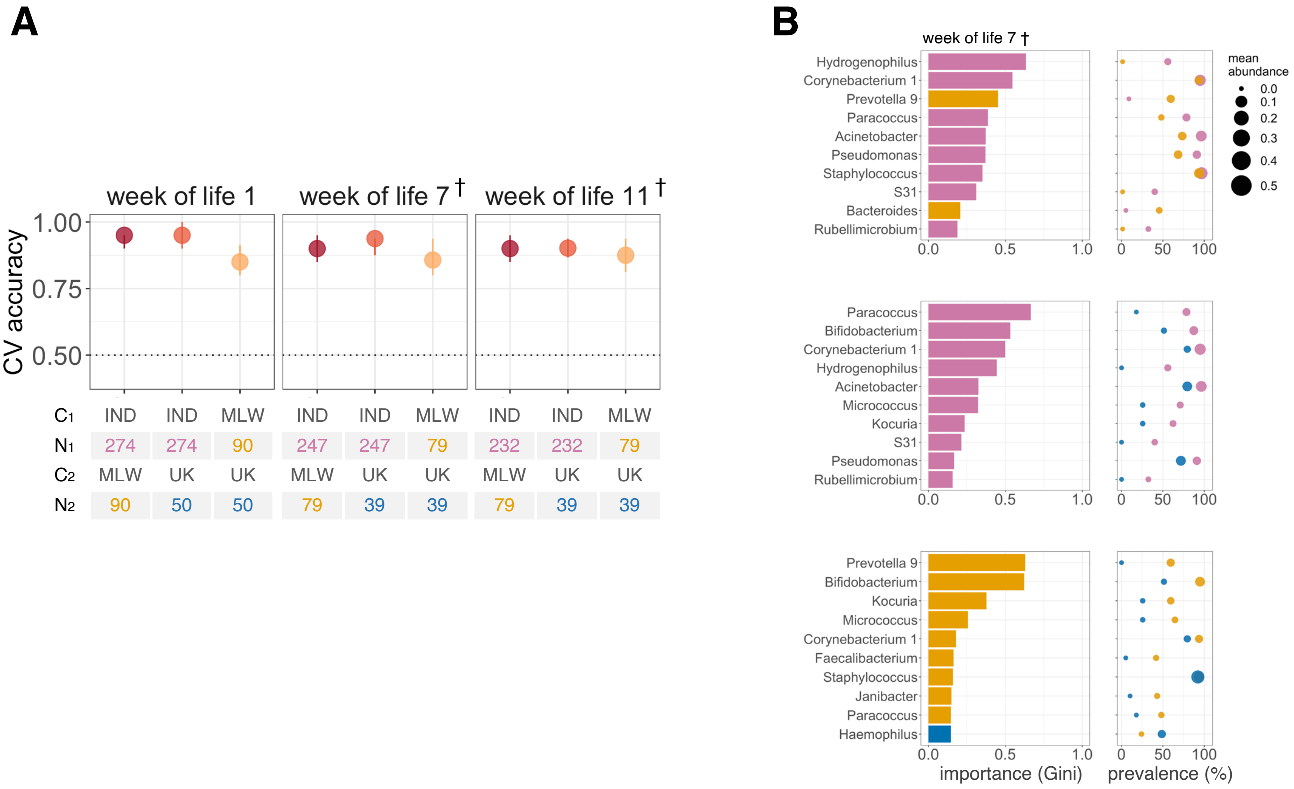

Supplementary Figure 5. Prediction of country by Random Forests. (A) Cross-validation accuracy of Random Forests. Median out-of-bag accuracy (proportion correctly assigned) and interquartile range across 20 iterations of 5-fold cross-validation are displayed. A random subset of 50 samples per country was used for each iteration. (**B**) The 10 most important genera selected by Random Forests for discriminating infants by country in the week after the first dose of oral rotavirus vaccine. Mean cross-validation importance scores based on Gini index are depicted alongside the prevalence and mean abundance of the corresponding genera. C, country; CV, cross-validation; IND, India; MLW, Malawi; †, +2 weeks in UK due to later vaccination schedule.

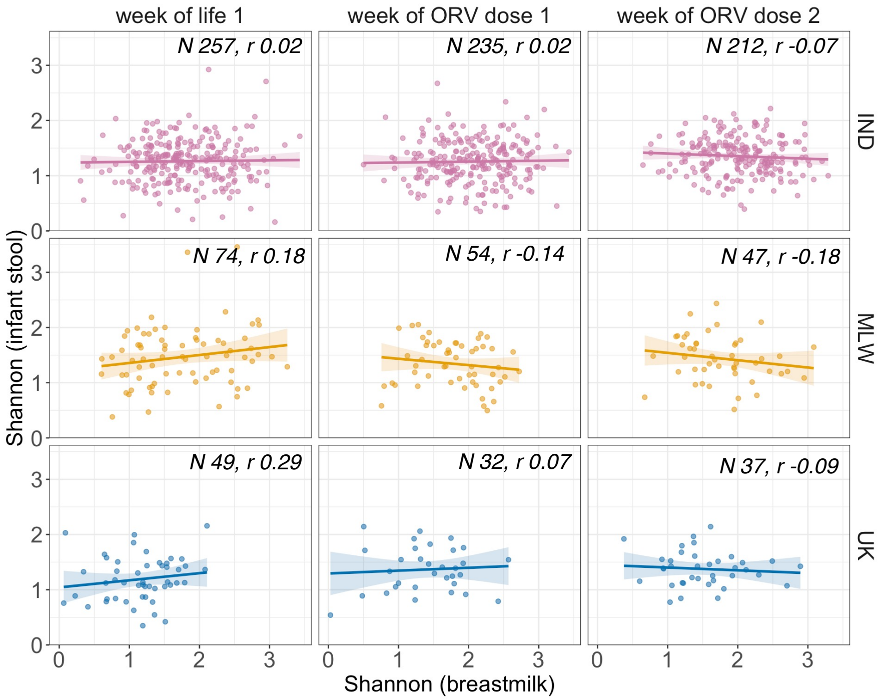

Supplementary Figure 6. Comparison of alpha diversity in paired breastmilk and infant stool samples. Genus-level Shannon index was calculated at a depth of 15,000 sequences per sample. Sample counts and Pearson's correlation coefficient (r) are indicated in italics. Stool samples were collected at the time of each ORV dose (weeks of life 6 and 10 in India and Malawi, weeks of life 8 and 12 in the UK), whereas breastmilk samples were collected 1 week after each dose. Lines show linear regression fits with 95% confidence intervals. *P* values were >0.05 for all comparisons.

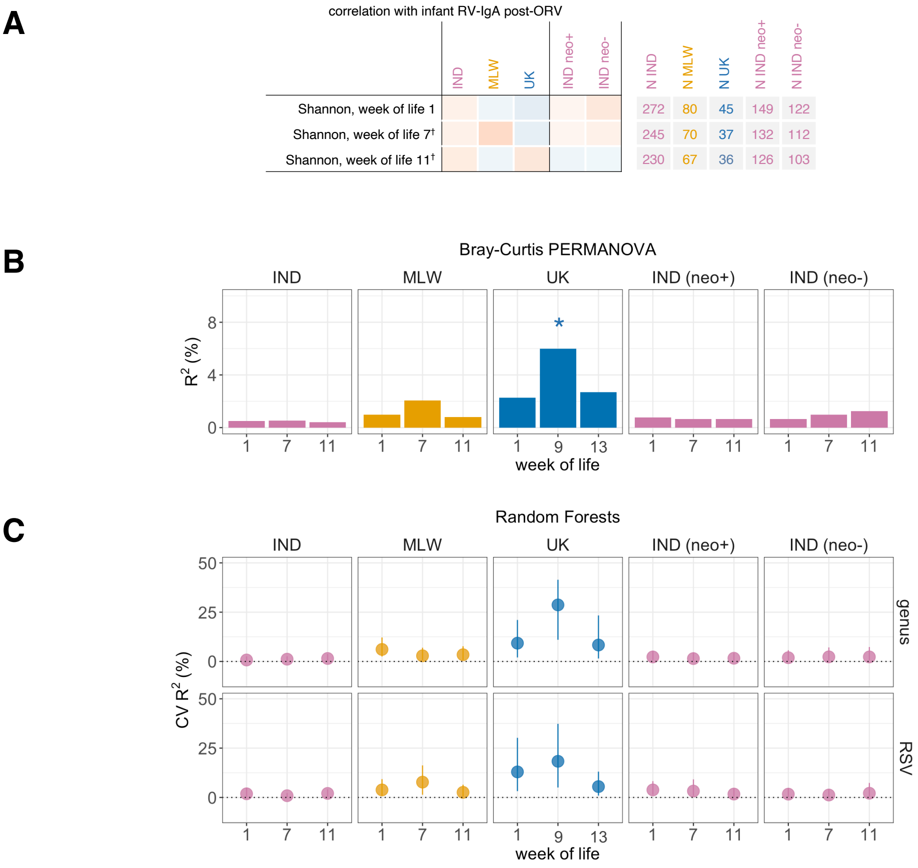

N per RF iteration	272	245	230	80	70	67	45	37	36	149	132	126	122	112	103
N genera included	152	140	129	143	141	131	77	102	88	152	142	123	149	137	127
N correlated with RV-IgA (Aldex2)	0	0	0	0	0	0	0	0	0	0	0	0	0	0	0
N RSVs included	394	315	301	326	359	338	194	294	269	407	349	294	357	309	295
N correlated with RV-IgA (Aldex2)	0	0	0	0	0	0	0	0	0	0	0	0	0	0	0

Supplementary Figure 7. Association between breastmilk microbiota composition and post-vaccination rotavirus-specific IgA concentration. (A) Analysis of alpha diversity, based on genus-level Shannon index. Shannon index was compared with log-transformed RV-IgA values using Pearson's correlation coefficient (*r*) with two-sided hypothesis testing. **(B)** Proportion of variation in microbiota composition associated with RV-IgA, calculated via PERMANOVA using genus-level unweighted Bray–Curtis distances. **(C)** Cross-validation accuracy of Random Forests for prediction of post-vaccination RV-IgA. Median out-of-bag R^2 and interquartile range are displayed for predicted vs observed RV-IgA across 20 iterations of 5-fold cross-validation. Correlations between log-ratio transformed taxon abundance counts and RV-IgA were determined via Aldex2 with two-sided Spearman's rank test. Taxa were classified as discriminant if they had an FDR-adjusted p value of <0.05. IND, India; MLW, Malawi; neo+, infected with rotavirus neonatally (defined by detection of rotavirus shedding in week of life 1 or baseline seropositivity); neo–, uninfected with rotavirus neonatally; ns, not significant; RF, Random Forests; RSV, ribosomal sequence variant; * $p < 0.05$.

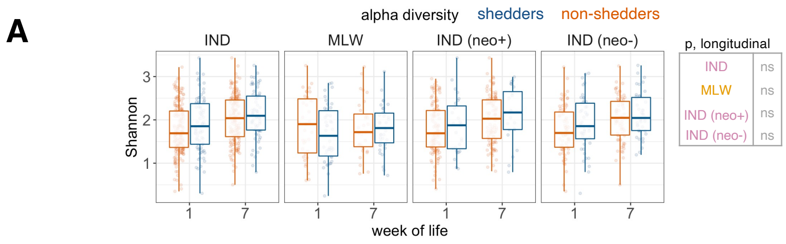

	IND 1	IND 7	MLW 1	MLW 7	IND (neo+) 1	IND (neo+) 7	IND (neo-) 1	IND (neo-) 7
N shedders	77	68	42	35	28	24	48	43
N non-shedders	195	177	35	32	119	106	74	69

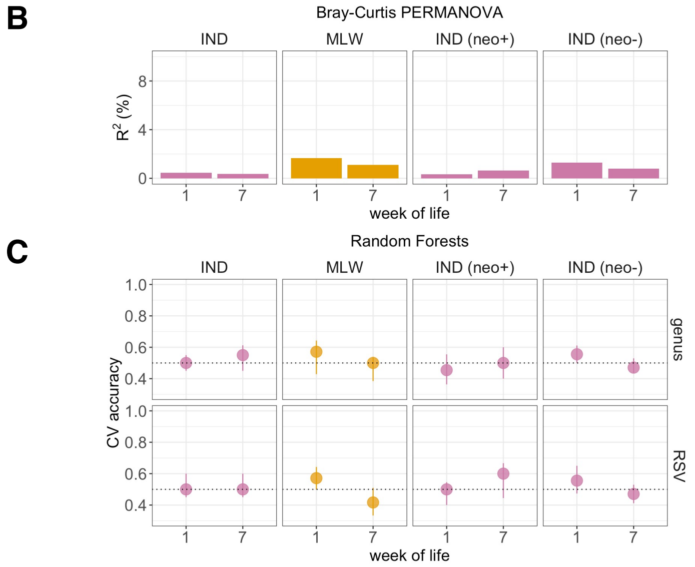

	IND 1	IND 7	MLW 1	MLW 7	IND (neo+) 1	IND (neo+) 7	IND (neo-) 1	IND (neo-) 7
N shedders	77	68	42	35	28	24	48	43
N non-shedders	195	177	35	32	119	106	74	69
N per RF iteration	100	100	70	64	56	48	96	86
N genera included	178	152	182	171	172	150	178	154
N differential genera	0	0	0	0	0	0	0	0
N RSVs included	518	402	549	547	550	512	519	396
N differential RSVs	0	0	0	0	0	0	0	0

Supplementary Figure 8. Association between breastmilk microbiota composition and dose 1 oral rotavirus vaccine shedding. See **Figure 3** for details; the same analyses of (A) alpha diversity, (B) beta diversity, and (C) Random Forests cross-validation accuracy are presented here with shedding 1 week after the first dose of oral rotavirus vaccine as outcome. Comparisons were not performed for the UK due to the small number of non-shedders (5 out of 60 infants) in this cohort. CV, cross-validation; IND, India; MLW, Malawi; neo+, infected with rotavirus neonatally (defined by detection of rotavirus shedding in week of life 1 or baseline seropositivity); neo−, uninfected with rotavirus neonatally; ns, not significant; RF, Random Forests; RSV, ribosomal sequence variant; *$p < 0.05$.
